# Comprehensive multi-omics analysis identifies chromatin regulator-related signatures and TFF1 as a therapeutic target in lung adenocarcinoma through a 429-combination machine learning approach

**DOI:** 10.3389/fimmu.2024.1481753

**Published:** 2024-10-30

**Authors:** Jun Fan, BoGuang Chen, Hao Wu, Xiaoqing Liang, Wen Shen, Xiaye Miao

**Affiliations:** ^1^ Department of Thoracic Surgery, The First Affiliated Hospital of Nanjing Medical University, Nanjing, China; ^2^ Oncology Department I, Huai’an 82 Hospital, Huai’an, Jiangsu, China; ^3^ Department of Oncology, The Affiliated Huai’an Hospital of Xuzhou Medical University and The Second People’s Hospital of Huai’an, Huai’an, Jiangsu, China; ^4^ Department of Oncology, Beijing Shijitan Hospital, Capital Medical University, Beijing, China; ^5^ Department of Respiratory Diseases, The Affiliated Huai’an Hospital of Xuzhou Medical University, The Second People’s Hospital of Huai’an, Huai’an, Jiangsu, China; ^6^ Department of Laboratory Medicine, Northern Jiangsu People’s Hospital, Yangzhou, China

**Keywords:** lung adenocarcinoma, machine learning, TFF1, multi-omics, chromatin regulator

## Abstract

**Introduction:**

Lung cancer is a leading cause of cancer-related deaths, with its incidence continuing to rise. Chromatin remodeling, a crucial process in gene expression regulation, plays a significant role in the development and progression of malignant tumors. However, the role of chromatin regulators (CRs) in lung adenocarcinoma (LUAD) remains underexplored.

**Methods:**

This study developed a chromatin regulator-related signature (CRRS) using a 429-combination machine learning approach to predict survival outcomes in LUAD patients. The CRRS model was validated across multiple independent datasets. We also investigated the impact of CRRS on the immune microenvironment, focusing on immune cell infiltration. To identify potential therapeutic targets, TFF1, a chromatin regulator, was knocked down using siRNA in LUAD cells. We assessed its impact through apoptosis analysis, proliferation assays, and in vivo tumor growth studies. Additional validation was performed using Ki67 expression and TUNEL assays.

**Results:**

The CRRS accurately predicted survival outcomes and was shown to modulate immune cell infiltration in the tumor microenvironment. High-risk patients demonstrated increased activity in cell cycle regulation and DNA repair pathways, along with distinct mutation profiles and immune responses compared to low-risk patients. TFF1 emerged as a key therapeutic target. Knockdown of TFF1 significantly inhibited LUAD cell proliferation, induced apoptosis, and suppressed in vivo tumor growth. Ki67 and TUNEL assays confirmed the role of TFF1 in regulating tumor growth and cell death.

**Discussion:**

These findings highlight the potential of chromatin regulators in prognostic modeling and immune modulation in LUAD. TFF1 was identified as a promising therapeutic target, suggesting that targeting TFF1 could provide new treatment strategies. Further research is warranted to explore its full potential and therapeutic applicability.

## Introduction

Lung adenocarcinoma (LUAD) represents a significant global health challenge, with its incidence steadily increasing (1, 2). While traditional treatments such as surgery and chemotherapy remain key options, recent advances in molecular biology and technology have provided new opportunities to identify molecular targets and develop targeted therapies for LUAD (3–5). Some patients with LUAD have specific genetic mutations, such as alterations in EGFR, ALK, and HER2, which allow them to benefit from targeted therapies (6, 7). Despite significant progress in innovative therapies, the overall survival rate post-diagnosis remains below 5%, driving continuous efforts to find more effective treatments and early detection methods (6, 8). However, despite advancements in treatment, the overall survival rate for LUAD remains below 5%, prompting ongoing research efforts to discover more effective therapies and improve early detection6,8. Resistance to current treatments is widespread, further emphasizing the need for the development and validation of new therapeutic strategies (9–11). The advent of immunotherapy has been a breakthrough in cancer treatment, yielding promising results (12, 13). However, not all patients respond equally to immunotherapy, and understanding this variability is a key challenge (14).

Epigenetics, first defined by Waddington in 1942 as the study of heritable changes that do not involve alterations to the DNA sequence (15, 16). Epigenetic processes primarily involve changes surrounding nuclear material, including the regulation of chromatin structure, nucleosome positioning, histone modifications, DNA methylation and demethylation, and interactions between enhancers and promoters (17–19). Epigenetic regulation is mediated by chromatin regulators (CRs), which are categorized into three main groups: DNA methylation regulators, histone modification regulators, and chromatin remodeling factors. Each group plays an indispensable role in epigenetic control (20, 21). In the realms of DNA methylation and histone modifications, CRs are further classified as readers, writers, and erasers. Readers recognize specific modifications on DNA or histones through unique domain structures, while writers and erasers are responsible for adding and removing these modifications, such as acetylation or deacetylation (22, 23). Chromatin remodeling functions include repositioning, ejecting, or altering the state of nucleosomes. The cumulative effects of CR-mediated epigenetic activities regulate DNA accessibility, influence polymerase transcriptional utilization, and thereby affect gene expression levels (24).

CRs are involved in numerous biological processes, including inflammation, memory, apoptosis, autophagy, and cancer development (25). By modulating chromatin structure, CRs can respond to both internal and external signals to regulate gene expression epigenetically. When CRs are mutated or misexpressed, they can cause widespread changes in the epigenetic landscape, leading to various diseases, including cancer (26, 27). Although much is known about the role of CRs in general cancer biology, their specific functions in LUAD and their impact on immunotherapy outcomes remain poorly understood, necessitating further research. A deeper understanding of CRs could lead to new insights into LUAD progression and novel therapeutic opportunities (28–30).

In this study, we employed an innovative artificial intelligence framework using 429 machine learning algorithms (31) and ten-fold cross-validation to develop a chromatin regulator-related signature (CRRS) based on TCGA-LUAD data. This CRRS was evaluated through analysis of both intrinsic and extrinsic immune landscapes using integrated multi-omics data, focusing on the expression patterns and prognostic significance of CRs in LUAD. We successfully constructed and validated a prognostic model based on 29 CRs, which accurately predicted survival outcomes for LUAD patients in both internal and external datasets, as well as pan-cancer analyses. Importantly, experimental validation demonstrated that silencing the CR TFF1 inhibited tumor growth and reduced the malignant behavior of LUAD cells *in vitro* and *in vivo*, indicating that TFF1 may be a promising therapeutic target for lung cancer treatment.

## Material and methods

### Data acquisition

Multi-omics data and clinical information relevant to LUAD were obtained from The Cancer Genome Atlas (TCGA) database (https://portal.gdc.cancer.gov), encompassing RNA sequencing data, mutational profiles, and survival outcomes. For the purpose of validating our model, an additional six datasets were procured from the Gene Expression Omnibus (GEO) database, including GSE42127, GSE31210, GSE30219 (32), GSE29016 (33), GSE26939 (34) and GSE13213 (35) (http://www.ncbi.nlm.nih.gov/geo).

A dataset, comprising normalized transcriptomic and genome across 33 The Cancer Genome Atlas (TCGA) cohorts, was obtained from the University of California Santa Cruz (UCSC) Xena database (https://xenabrowser.net) to identify the predictive ability of our signature for pan-cancer.

To maintain consistency in data formatting from the onset of analysis, all datasets were subjected to log2 transformation. In order to address the possibility of batch effects, the *‘ComBat’* function within the *‘sva’* package for R was employed (36).

### Different expression and enrichment analysis

Chromatin-related genes are derived from FACER database (37). The *‘limma’* package was utilized to identify differentially expressed chromatin regulators (DECRs) between LUAD specimens and normal lung tissue, applying a significance criterion of P. adjust < 0.05 and an absolute log_2_ fold change (log2FC) of 1 or greater. To decipher the biological implications of the DECRs, we performed Kyoto Encyclopedia of Genes and Genomes (KEGG) and Gene Ontology (GO) enrichment analyses with the aid of the *‘clusterProfiler’* package in R (38).

### Development of signatures using an artificial intelligence network

We endeavored to establish a precise and robust CRRS for the prognostication of LUAD patient outcomes. To accomplish this, we orchestrated an extensive artificial intelligence framework comprising 429 combined algorithms, incorporating 27 varied algorithms from domains of traditional regression, machine learning, and deep learning. These algorithms included stepwise Cox, random survival forest (RSF), gradient boosting machine (GBM), supervised principal components (SuperPC), oblique random survival forests (obliqueRSF), conditional random forests (CForest), gradient boosting with component-wise linear models (GLMBoost), gradient boosting with regression trees (BlackBoost), recursive partitioning and regression trees (Rpart), parametric survival model (Survreg), Ranger, conditional inference trees (Ctree), least absolute shrinkage and selection operator (LASSO), partial least squares regression for Cox (plsRcox), survival support vector machine (survival-SVM), Ridge, elastic network (Enet), deephit survival neural network (DeepHit), deepsurv survival neural network (DeepSurv), cox-time survival neural network (CoxTime), extreme gradient boosting (XGBoost), Boruta, logistic-hazard survival neural network (Logistic-Hazard), PC-hazard survival neural network (PC-hazard), akritas conditional non-parametric survival estimator (Akritas), Coxboost, and variable selection oriented LASSO bagging algorithm (VSOLassoBag). Within the TCGA dataset, we harnessed these 429 distinctive algorithmic combinations to create predictive models, evaluating the predictive performance of each combination through the concordance index (C-index) across all cohorts. The selection of the supremely algorithmic combination was determined by yielding the highest average C-index. The source code and specific parameters for the artificial intelligence network are available at the following GitHub repository: https://github.com/Xulab2024/ML.

### Functional annotation of the CRRS

Gene set variation analysis (GSVA) and gene set enrichment analysis (GSEA) were conducted leveraging the Molecular Signatures Database (MSigDB) (39). These analyses were facilitated by employing the ‘GSVA’ and ‘clusterProfiler’ packages in R (40). Additionally, *Metascape* was utilized for further enrichment analysis (41).

### Immune infiltration analysis

Based on the CRRS scores, divide the samples into high-risk and low-risk groups. Collect and organize RNA-seq expression data from TCGA-LUAD and other relevant datasets. Estimate immune cell infiltration using tools such as CIBERSORT, xCell, and EPIC. Combine the estimation results into a comprehensive immune cell infiltration matrix and visualize it using the *“ComplexHeatmap”* package. We also acquired 29 classical immune signatures from the work of He et al. (42). The cytolytic activity scores (CYTs) were estimated using the geometric mean of GZMA and PRF1 (43). Aneuploidy scores were defined as the sum total of the amplified or deleted (collectively, “altered”) arms (44). TCR diversity scores (Shannon entropy and richness) and BCR diversity scores (Shannon entropy and richness) were inferred from cancer RNA-seq data (44).

### Cell culture

LEWIS and TE1 cell lines were obtained from the American Tissue Culture Collection (ATCC). Both LEWIS and TE1 cell lines were maintained in RPMI 1640 medium supplemented with 10% Fetal Bovine Serum (FBS) (Gibco, USA) and 1% penicillin/streptomycin solution. All cells were cultured at 37°C with 5% CO_2_.

### Cell Counting Kit-8

Seed the test cells at a density of 3000 cells per well in a 96-well plate and incubate at 37°C with 5% CO2 for 24 hours to allow cell adhesion. At 0h, 24h, 48h, and 72h, add 10 µL of CCK-8 solution to each well. After incubating for 2 hours, measure the absorbance at 450 nm using a microplate reader to assess cell proliferation. Compare the absorbance values with the control group for data analysis. Ensure the entire process is conducted under sterile conditions to guarantee the accuracy of the results.

### SiRNA transfection

Seed cells in a 6-well plate, aiming for 70% confluence at the time of transfection. Dilute the siRNA and transfection reagent separately in serum-free medium, mix them, and incubate for 10-20 minutes to form the complex. Add the complex to the cell culture medium, gently swirl to ensure even distribution, and incubate overnight at 37°C with 5% CO2. After transfection, replace with serum-containing medium and continue to incubate for 24-72 hours. Perform qPCR to evaluate gene knockdown efficiency. The target sequences are listed in Appendix 2.

### Colony formation assay

Seed the test cells at a density of 500 cells per well or dish in a 6-well plate or 10 cm culture dish, gently swirl to ensure even distribution, and incubate at 37°C with 5% CO2 for 14 days until visible colonies form. After incubation, discard the medium, gently wash the cells with PBS 2-3 times, fix the cells with 4% paraformaldehyde for 20 minutes, and then stain with crystal violet for 30 minutes. Rinse with running water to remove excess stain, and once the background is clear, count the number of colonies.

### Qrt-PCR

Total RNA was extracted using TRIzol reagent (Invitrogen) according to the manufacturer’s instructions. RNA concentration and purity were measured using a NanoDrop 2000 spectrophotometer (Thermo Scientific). Genomic DNA contamination was removed by DNase I treatment (Invitrogen). One microgram of total RNA was reverse-transcribed into cDNA using the PrimeScript RT Reagent Kit (Takara). qRT-PCR was performed using SYBR Premix Ex Taq (Takara) on an ABI 7500 Fast Real-Time PCR System (Applied Biosystems) with the following cycling conditions: 95°C for 30 seconds for initial denaturation, followed by 40 cycles of 95°C for 5 seconds, 60°C for 34 seconds for annealing and extension. Gene expression for each sample was normalized to the internal control gene (e.g., GAPDH), and data were analyzed using the 2^(-ΔΔCt) method. The primer sequences used are listed in **Supplementary Table S1**.

### Flow cytometry for apoptosis detection

After digesting and collecting the cells for analysis, wash them once with sterile, pre-cooled PBS. Then stain the cells with Annexin V and 7-AAD, incubating in the dark for 20 minutes. Subsequently, perform detection using a flow cytometer, and finally, analyze the data using FlowJo software.

### Subcutaneous tumor model in mice

Animal experiments were approved by the Ethics Committee of the Affiliated Huai’an Hospital of Xuzhou Medical University. Lewis cells were cultured and resuspended in serum-free medium, adjusting the cell concentration to 5×10^5^ cells/100 µL. Using a sterile syringe, 100 µL of the cell suspension was injected subcutaneously into the right dorsal flank of 6-8 weeks-old female C57BL/6 mice. After injection, the mice were monitored for health status, and tumor volume was measured regularly until the experimental endpoint, at which point the mice were euthanized by cervical dislocation.

### Immunohistochemical staining for Ki67

Tissue sections were first deparaffinized and rehydrated through a graded series of alcohols. Antigen retrieval was then performed using a suitable buffer, followed by blocking with a serum to reduce non-specific binding. The sections were incubated with a primary antibody against Ki67 overnight at 4°C. After washing, a secondary antibody conjugated to an enzyme was applied, followed by a chromogenic substrate to visualize the staining. The sections were then counterstained with hematoxylin, dehydrated, and mounted for microscopic examination. The presence of Ki67-positive cells was evaluated as an indicator of cell proliferation.

### Fluorescent TUNEL staining on paraffin sections

First, paraffin sections are deparaffinized and rehydrated. Next, antigen retrieval is performed using proteinase K treatment. According to the TUNEL assay kit instructions, the TUNEL reaction mixture is then applied to the sections, followed by incubation to label DNA breaks. After staining, the results are observed under a fluorescence microscope, with positive signals indicating apoptosis.

### Statistical analysis

All data are expressed as mean ± standard error of the mean (SEM). Statistical analysis was performed using GraphPad Prism 8.0 and R 4.2.0 software. Comparisons between groups were made using one-way analysis of variance (ANOVA) or t-test, depending on the specific experimental design. When data followed a normal distribution, a t-test was used; for multiple group comparisons, ANOVA was employed. Statistical significance was determined by the P-value, with P < 0.05 considered significant. Significance levels are indicated by asterisks: *P < 0.05, **P < 0.01, ***P < 0.001, ****P < 0.0001.

## Results

### Variant landscape of chromatin regulator genes in LUAD patients

**Figure 1:**
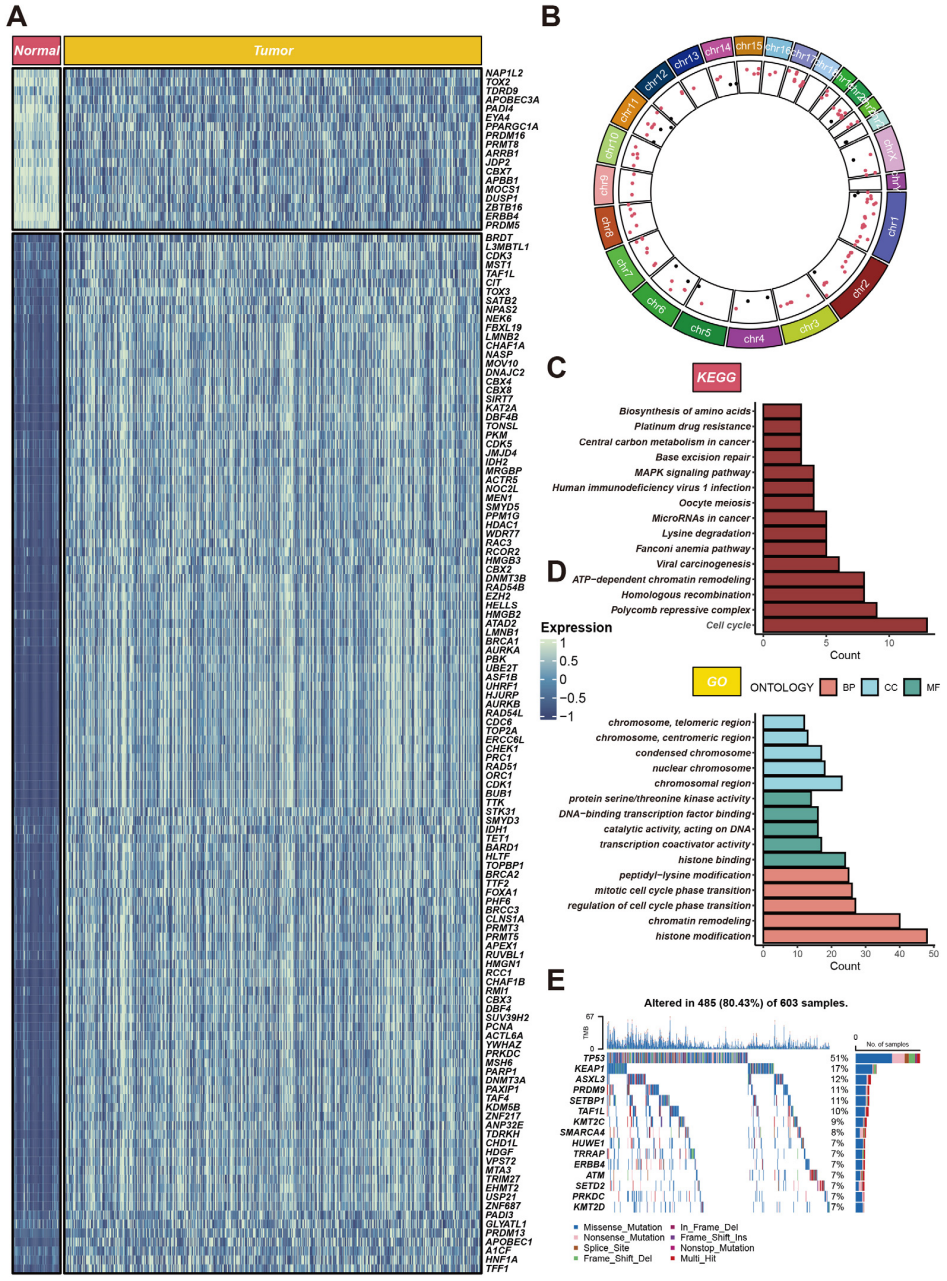
Expression profiles of CRs in LUAD. **(A)** Heatmap of differentially expressed CRs between cancerous and adjacent tissues in LUAD patients. **(B)** Circular plot of genomic variations. The circular plot illustrates gene variations across different chromosomes. **(C, D)** Gene function enrichment analysis. The upper part shows KEGG pathway analysis results, highlighting the main pathways enriched for CRs. **(E)** Gene mutation profile. It shows the mutation types and frequencies of CRs in 603 LUAD samples.

Within the TCGA-LUAD cohort, our study identified 134 differentially expressed genes (DEGs), all meeting the criteria of adjusted P < 0.05, and absolute log2 fold change (log2FC) exceeding 1. Of these, 116 genes were found to be upregulated, while 18 were downregulated in the LUAD group compared to non-tumor tissues. The standardized RNA expression levels of these DEGs are depicted as heatmaps in **Figure 1A**. Additionally, **Figure 1B** delineates the chromosomal locations of each DEG. Moreover, enrichment analyses conducted using Kyoto Encyclopedia of Genes and Genomes (KEGG) and Gene Ontology (GO) revealed the implication of these DEGs in a spectrum of biological pathways, notably cell cycle, polycomb repressive complex, histone modification, chromatin remodeling (**Figures 1C, D**). We also scrutinized chromatin regulator gene alterations in LUAD patients within the TCGA cohort, uncovering that approximately 80.43% (485 out of 603) of the individuals harbored mutations in these genes. The top 15 mutations within chromatin regulator genes are outlined, with TP53 registering the highest mutation frequency at 51%, and the remaining fourteen variations ranging between 7% and 17% in prevalence (**Figure 1E**).

### CRRS construction and validation

**Figure 2:**
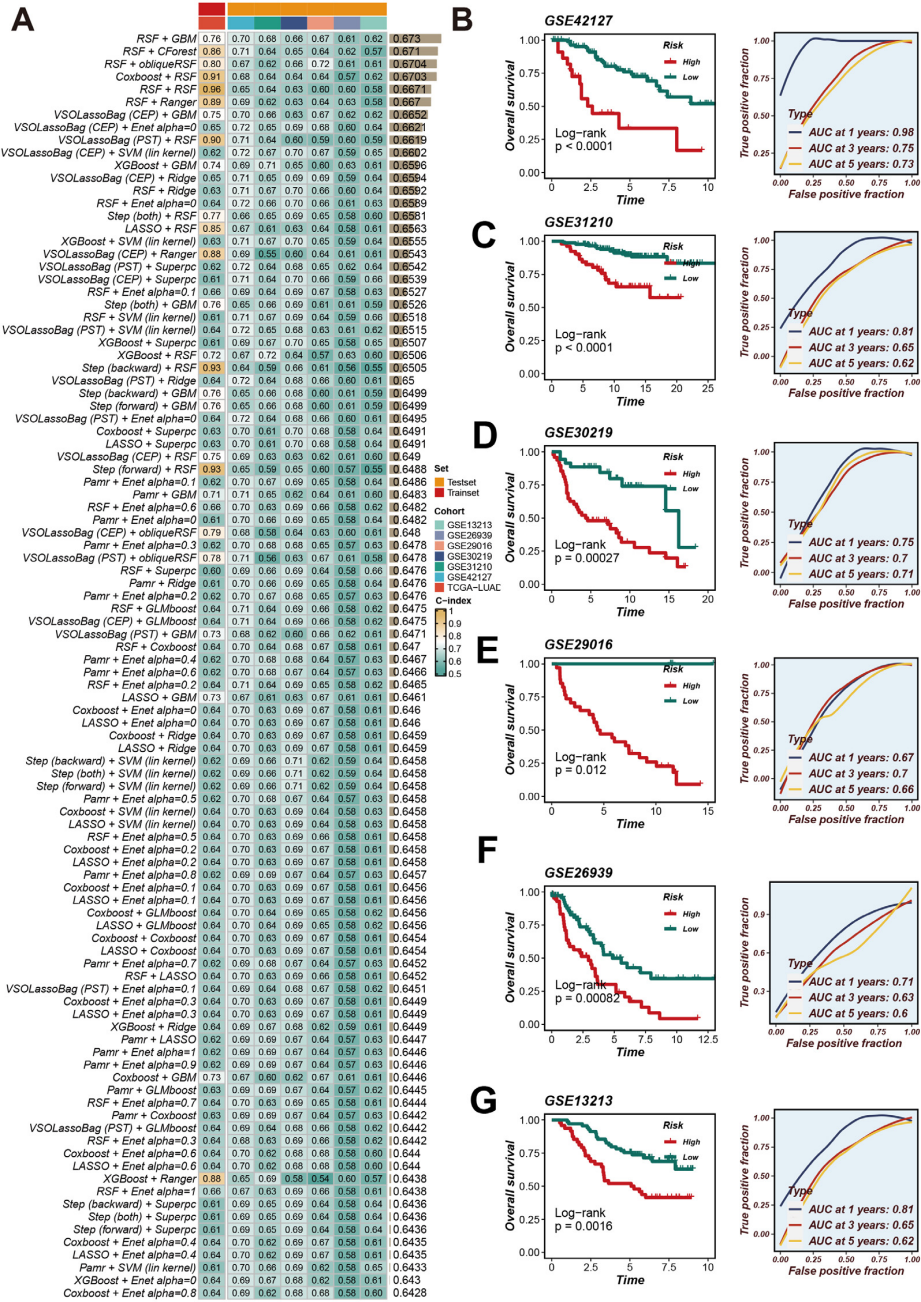
CRRS was developed and validated using multiple machine learning algorithms. **(A)** Using a 10-fold cross-validation framework, a total of 429 combinations of machine learning algorithms were employed, and the c-index for each model was calculated. **(B–G)** Kaplan-Meier survival analysis and time-dependent ROC curves for 1-year, 3-year, and 5-year OS in the high- and low-risk groups based on the optimal cut-off value of CRRS in the GSE42127, GSE31210, GSE30219, GSE29016, GSE26939, and GSE13213 datasets.

We collated and analyzed survival data of LUAD patients, applying univariate Cox regression analysis to initially screen for genes associated with survival. Within the TCGA-LUAD cohort, we identified 29 genes that satisfied the significance threshold of P < 0.05. Following this, we explored 429 algorithmic combinations within the TCGA-LUAD cohort and computed the concordance index (C-index) for each model across the respective cohorts. The integration of RSF and GBM produced the most exemplary average C-index of 0.673. This led us to adopt this integrated approach as our finalized CRRS (**Figure 2A**). Employing the optimal cut-off for the CRRS enabled the stratification of LUAD patients into distinct high- and low-risk groups. Observations pointed to a notable disparity in survival, with high-risk patients demonstrating significantly poorer overall survival (OS) in comparison to their low-risk counterparts across all cohorts (P < 0.05), as evidenced by **Figures 2B–G**. Furthermore, the application of time-dependent Receiver Operating Characteristic (ROC) analyses validated the prognostic accuracy of our scoring method consistently across all patient cohorts (illustrated in **Figure 2B**).

### Comparison of CRRS with other clinical features

**Figure 3:**
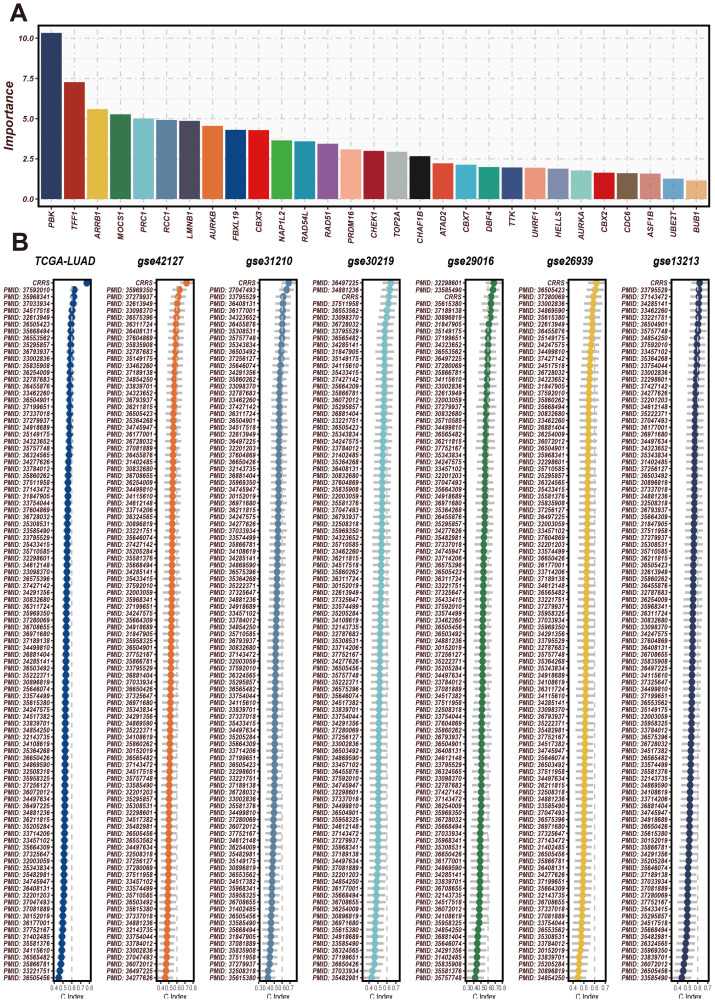
The significance of CRRS across various datasets and analyses. **(A)** The importance scores of the 29 CRRS-related genes used in the model. The horizontal axis represents gene names, and the vertical axis represents importance scores. PBK has the highest importance score, followed by TFF1, AR3BP1, and others. These genes have high predictive ability and discriminatory power in the prediction model. **(B)** C-index of CRRS in TCGA-LUAD and multiple GEO datasets (including GSE42127, GSE31210, GSE30219, GSE29016, GSE26639, GSE13213). In each dataset’s scatter plot, the horizontal axis represents the C-index value, and the vertical axis represents the PMID number of the cited literature.

Initially, CRRS was compared with other clinical features (age, gender, EGFR status, KRAS status, p53 status, stage, T staging, smoking status). The results revealed that the C-index values of CRRS were higher than those of other clinical features, consistently across in the validation cohorts (**Figure 3A**). Subsequently, SRS was compared with 101 predictive signatures from published studies, and the results demonstrated that the SRS exhibited the best predictive performance across all seven datasets (**Figure 3B**).

**Figure 4:**
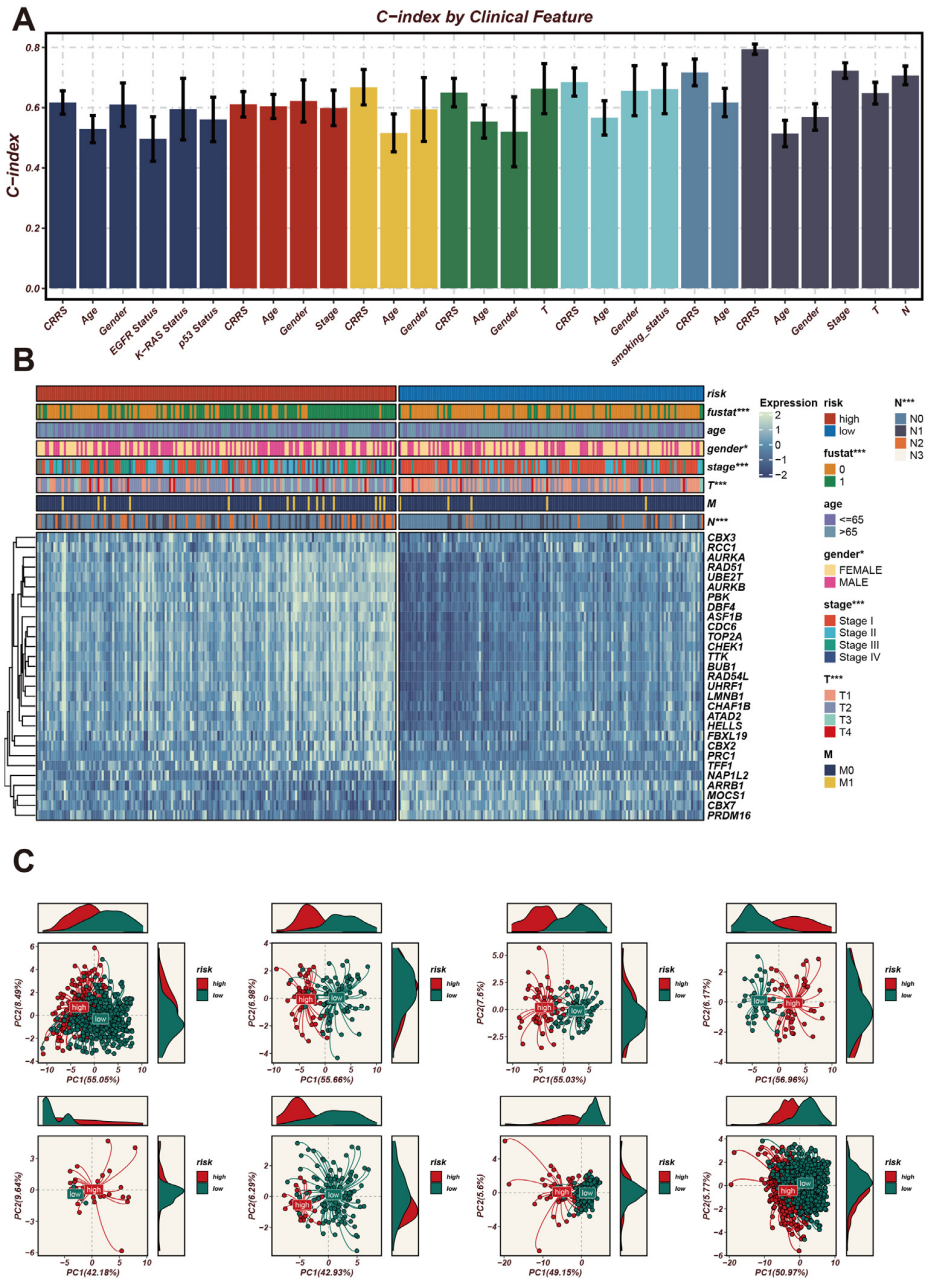
Expression patterns of CRRS under different clinical characteristics and their impact on the model’s predictive ability. **(A)** A bar chart of the C-index for clinical characteristics. The horizontal axis represents different clinical characteristics, and the vertical axis represents C-index values, which are used to assess the accuracy of the model’s predictive ability. **(B)** Heatmap of CRRS expression in high- and low-risk groups with clinical characteristics annotations. The heatmap shows the expression levels of CRRS genes across different samples. The annotation bar at the top indicates the clinical characteristics of the samples, including risk level, age, gender, stage, T stage, and M stage. **(C)** Principal Component Analysis (PCA) scatter plot. It shows the distribution of high-risk and low-risk group samples on the first two principal components (PC1 and PC2). *p < 0.05, ***p < 0.001.

The C-index (Concordance Index) is a metric used to evaluate the predictive accuracy of a survival model, where values closer to 1 indicate better model performance. Next, we analyzed the expression patterns of CRRS across different clinical features. The C-index (Concordance Index) is a metric used to evaluate the predictive accuracy of a survival model, where values closer to 1 indicate better model performance. As shown in **Figure 4A**, we used the C-index to assess the predictive accuracy of the CRRS model. Several clinical features, including CRRS, age, gender, stage, T stage, and N stage, exhibited high C-index values exceeding 0.6, indicating that these features have good predictive performance. Next, we used a heatmap to illustrate the expression profiles of CRRS utilized for modeling in high-risk and low-risk groups (**Figure 4B**). The gene expression profiles of high-risk patients are distinct from those of low-risk patients, indicating the potential of these genes as biomarkers for risk stratification in cancer prognosis.

Subsequently, PCA plots were employed to display the distribution of high-risk and low-risk groups in the principal component space (**Figure 4C**). The high-risk and low-risk groups formed distinct clusters, demonstrating that the principal components effectively captured the differences between these groups. The consistent clustering patterns across different principal component analyses further validate the robustness of the CRRS model in risk stratification.

### The expression profile of CRRS across different cancer types

**Figure 5:**
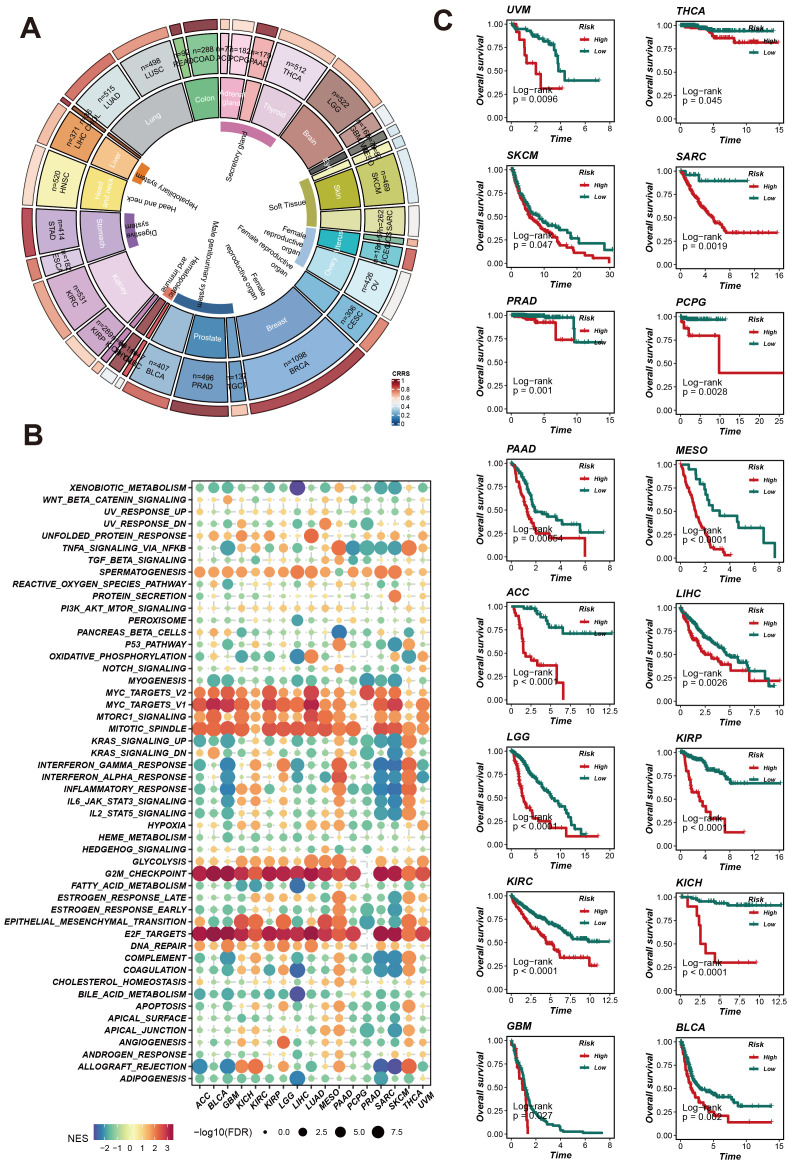
CRRS predicts pan-cancer patient survival and impacts potential biological pathways. **(A)** Pan-cancer CRRS Score Circle Plot: The circular plot illustrates the CRRS scores across different cancer types. **(B)** Pan-cancer Gene Set Enrichment Analysis (GSEA) Bubble Plot. **(C)** Survival Analysis Curves for Different Cancer Types: Kaplan-Meier curves display the overall survival rates of patients in high-risk and low-risk groups.

To assess and enhance the predictive capability of CRRS, we evaluated its survival prediction power across various cancer types. We analyzed the expression patterns of CRRS in different cancers, the enrichment of related signaling pathways, and its impact on patient survival. The ring chart in **Figure 5A** shows the expression levels of CRRS across various cancer types. Significant differences in CRRS expression levels were observed among different cancer types, suggesting that CRRS may play diverse roles in the occurrence and development of these cancers. The GSEA enrichment analysis of pan-cancer results (**Figure 5B**) indicate that CRRS may be involved in several key signaling pathways, including the MYC-TARGETS, G2M_CHEC KPOINT, E2F TARGETS etc, potentially influencing cancer progression. Furthermore, Kaplan-Meier survival curves (**Figure 5C**) demonstrate that in multiple cancer types, patients in the high-risk group have significantly lower survival rates compared to those in the low-risk group (such as UVM, THCA, SKCM, PRAD, etc.). This finding further validates the importance of CRRS in prognosis evaluation. These comprehensive analyses highlight the potential of CRRS as a prognostic biomarker across various cancers, providing crucial insights for clinical diagnosis and treatment.

### CRRS involvement in remodeling the LUAD immune microenvironment

**Figure 6:**
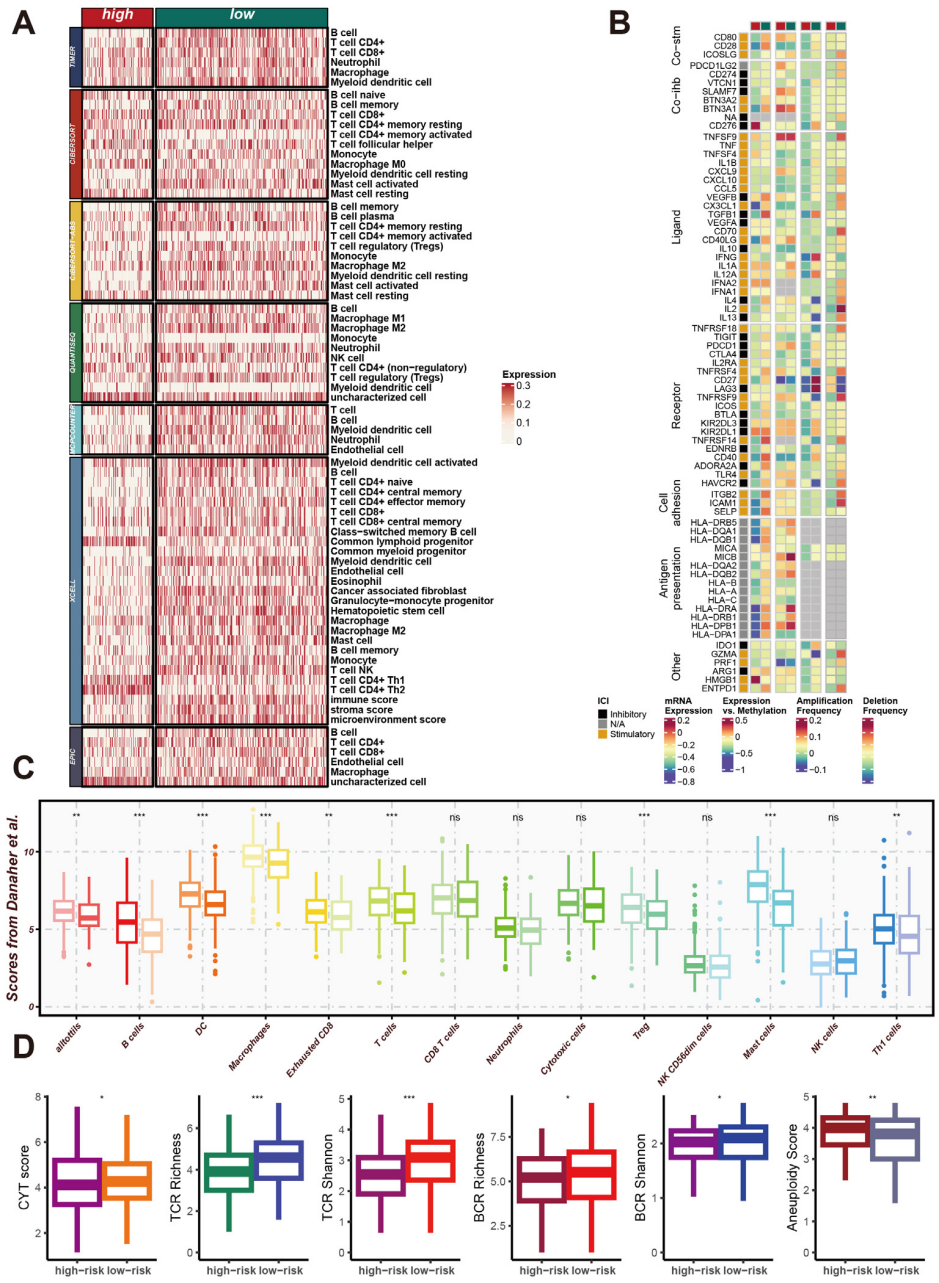
CRRS involvement in impacting the immune microenvironment of LUAD. **(A)** Heatmap of Differential Immune Cell Infiltration. This heatmap shows the expression levels of different immune cell types in high-risk and low-risk group samples. Immune cell types listed include T cells, B cells, macrophages, dendritic cells, and others. **(B)** Heatmap of Immune-Related Gene Expression. This heatmap displays the expression levels of immune-related genes in high-risk and low-risk group samples, including core cell surface molecules (Core-fab), ligands, receptors, cell antigens, and others. **(C)** Box Plot of Immune Scores. This box plot shows the distribution of various immune scores in high-risk and low-risk groups, including the immune score, stroma score, and microenvironment score. **(D)** Box Plot of Immune Diversity Scores. This box plot shows the distribution of various immune diversity scores in high-risk and low-risk group samples, including CYT score, TCR richness, TCR diversity (TCR Shannon), BCR richness, BCR diversity (BCR Shannon), and aneuploidy score. *p < 0.05, **p < 0.01, ***p < 0.001.

To explore the impact of CRRS on the LUAD immune microenvironment, we utilized seven algorithms—TIMER, CIBERSORT, CIBERSORT-ABS, QUANTISEQ, MCPCOUNTER, XCELL, and EPIC—to calculate the effect of CRRS on the tumor microenvironment (TME) of LUAD patients in high-risk and low-risk groups. As shown in **Figure 6A**, the heatmap displays the expression levels of various immune cell types, immune-related genes, and scores across high-risk and low-risk groups of LUAD patients. Significant differences in the expression of immune cell types, including T cells, B cells, macrophages, dendritic cells, and other immune cell subsets, were observed between the two groups. Additionally, the expression levels of immune-related genes also varied significantly between high-risk and low-risk patients. These genes are categorized into core surface molecules, ligands, receptors, cell antigens, and other categories, indicating distinct immune landscape characteristics between the two groups. Additionally, **Figure 6B** represents the expression levels of immune-related genes, specifically immune checkpoints. These genes are categorized into core surface molecules, ligands, receptors, cell antigens, and other categories. The variations in their expression levels between high-risk and low-risk patients indicate distinct immune landscape characteristics, with significant differences in immune checkpoint expression.

Moreover, the **Figure 6C** show the distribution of various immune scores, including immune score, stromal score, and microenvironment score, demonstrating distinct differences between high-risk and low-risk patients. **Figure 6D** illustrate the distribution of immune diversity scores such as CYT score, TCR richness, TCR Shannon, BCR richness, BCR Shannon, and Aneuploidy score, further supporting the differential immune profiles between the two groups. These results collectively demonstrate that CRRS significantly contributes to the remodeling of the immune microenvironment in LUAD, particularly affecting the expression of immune checkpoints, and potentially influencing the immune response and disease progression.

### Comprehensive analysis of CRRS and molecular pathways in LUAD

**Figure 7:**
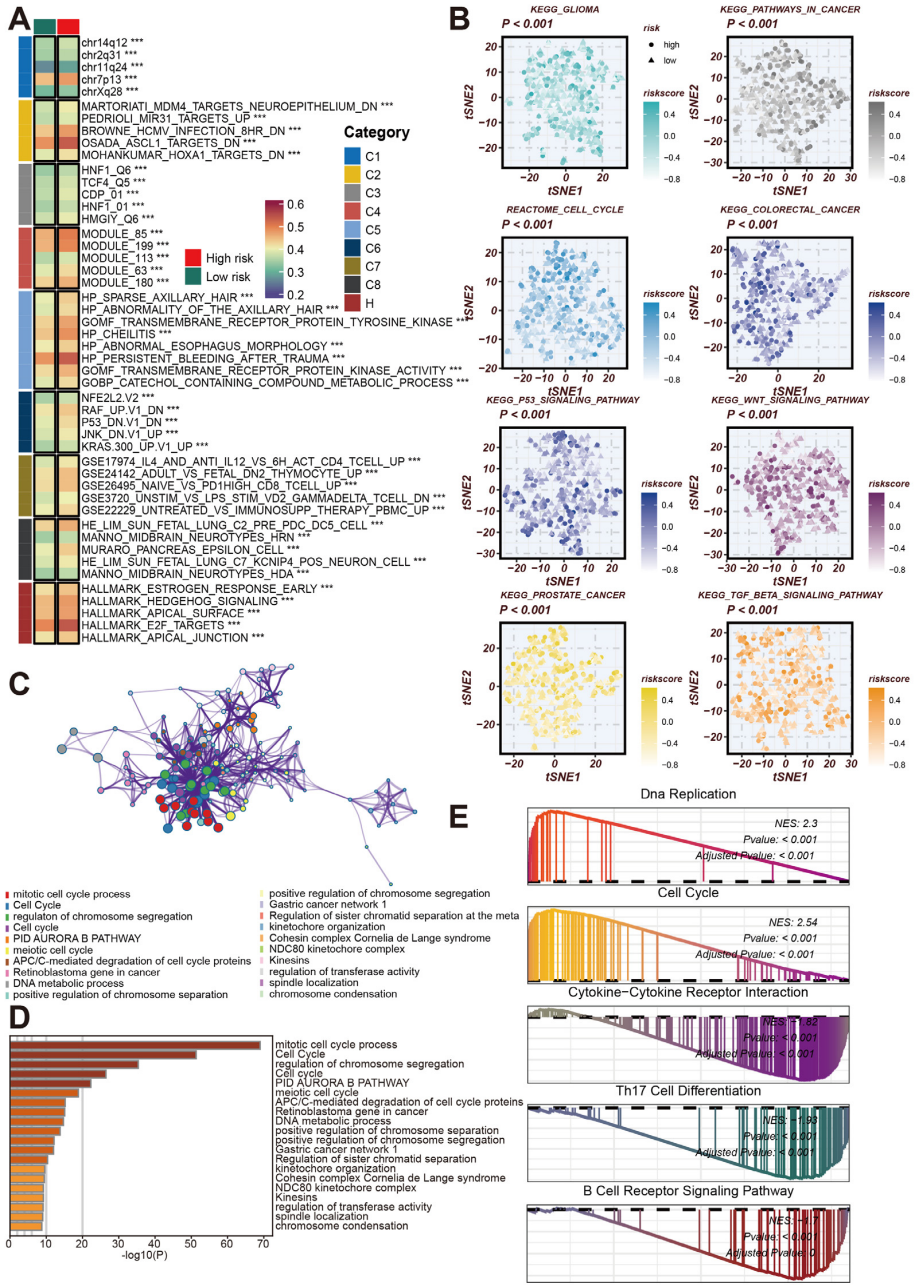
Analysis of the role of CRRS in regulating LUAD-related biological pathways. **(A)** GSVA Enrichment Analysis Heatmap. This heatmap shows the enrichment of different gene sets in high-risk and low-risk groups. **(B)** This tSNE plot shows the distribution of high-risk and low-risk group samples across different signaling pathways, including KEGG glioma, cell cycle, colorectal cancer, p53 signaling pathway, WNT signaling pathway, prostate cancer, TGF-β signaling pathway, and others. **(C)** Co-expression network related to the cell cycle and chromatin remodeling processes regulated by CRRS. **(D)** Bar chart of gene enrichment in biological processes and signaling pathways regulated by CRRS in LUAD. **(E)** Gene Set Enrichment Analysis (GSEA) Mountain Plot. This plot shows the enrichment of highly expressed genes in high- and low-risk groups across gene sets such as DNA replication, cell cycle, cytokine-cytokine receptor interaction, Th17 cell differentiation, and B cell receptor signaling pathway. ***p < 0.001.

Next, we conducted a more comprehensive analysis of the impact of CRRS on the biological behavior of LUAD. This analysis focused on the Cancer Risk-Related Score (CRRS) and its association with various molecular characteristics and biological pathways. The goal was to elucidate the pathways and processes significantly altered in high/low-risk LUAD patients, thereby providing insights into potential therapeutic targets and prognostic markers. The GSVA enrichment analysis displayed a heatmap of the differential expression of various molecular modules between high-risk and low-risk tumor samples. The categories on the right denote distinct functional modules. This heatmap reveals significant differences in the expression profiles of these modules, highlighting the molecular heterogeneity associated with tumor risk (**Figure 7A**).

Subsequently, we utilized KEGG and Reactome enrichment analyses to visualize the correlation between CRRS and risk stratification in LUAD patients, presenting these findings in t-SNE plots (**Figure 7B**). **Figure 7C** shows a network representation of biological processes and functional modules, with node colors representing different functional categories. This network illustrates the complex interactions and regulatory mechanisms involved in LUAD, emphasizing the interconnected nature of biological pathways.

To quantify the importance of these biological processes, **Figure 7D** features a bar graph depicting the results of gene function enrichment analysis. This analysis highlights significant biological processes such as the mitotic cell cycle process and c,GSEosome segregation regulation, which are crucial for tumor development and progression. The Gene Set Enrichment Analysis (GSEA) results for high-risk samples identified significant enrichment in pathways related to DNA Replication, Cell Cycle, Cytokine-Cytokine Receptor Interaction, Th17 Cell Differentiation, and B Cell Receptor Signaling Pathway (**Figure 7E**). These findings suggest that pathways associated with the cell cycle and DNA replication are markedly active in high-risk samples and imply potential roles for cytokine and immune-related pathways.

Overall, these analyses reveal that CRRS is involved in regulating molecular and pathway changes associated with LUAD, providing a foundation for further research into targeted therapies and prognostic indicators.

### *In vitro* and *in vivo* experiments validated that TFF1 knockdown inhibits the malignant phenotype of lung cancer cells

**Figure 8:**
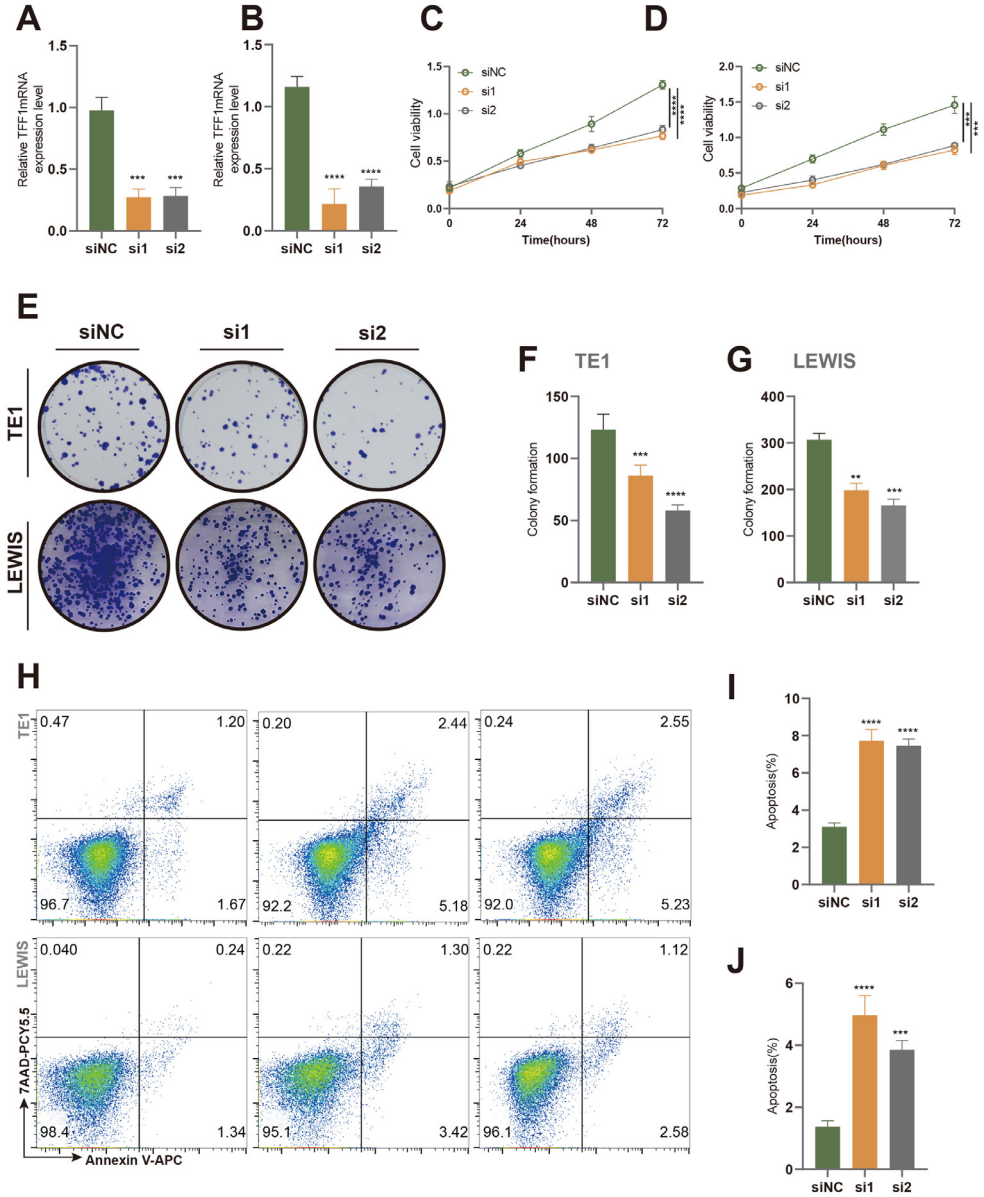
Effects of TFF1 knockdown on proliferation and apoptosis in TE1 and Lewis cell lines. **(A, B)** Relative TFF1 mRNA expression levels in TE1 **(A)** and Lewis **(B)** cell lines were measured by qRT-PCR after knockdown with two different siRNAs (si1 and si2), with siNC as the negative control. **(C, D)** Cell proliferation in TE1 **(C)** and Lewis **(D)** cells was assessed using the CCK-8 assay. **(E)** Colony formation assay showed a significant reduction in colony-forming ability in TE1 and Lewis cells after TFF1 knockdown. **(F, G)** Quantification of colony numbers in TE1 **(F)** and Lewis **(G)** cells. **(H)** Flow cytometry analysis of apoptosis in TE1 and Lewis cells stained with Annexin V-FITC and 7-AAD. **(I, J)** Quantification of the percentage of apoptotic cells in TE1 **(I)** and Lewis **(J)** cells. Data are presented as mean ± standard deviation. **p < 0.01, ***p < 0.001, ****p < 0.0001 indicate statistically significant differences compared to the siNC group.

Based on a comprehensive literature review and model C-index scoring, the TFF1 gene emerged as one of the top candidates, with an “Importance” score second only to PBK, underscoring its critical role in the model’s predictive outcomes. TFF1 has been documented to play a pivotal role in the progression of certain cancer types, including pancreatic cancer and gastric neoplasia, with its expression levels closely associated with tumor progression (45–48). Consequently, we selected TFF1 for experimental validation. Using SiRNA interference technology, we successfully knocked down TFF1 in murine-derived lung cancer cells (LEWIS) and human lung cancer cells (TE1) (**Figures 8A, B**). The results demonstrated that TFF1 knockdown significantly inhibited cell viability (**Figures 8C, D**) and colony formation (**Figures 8E–G**) in both cell lines. Moreover, flow cytometry analysis revealed a marked increase in apoptosis rates in TFF1 knockdown cells compared to controls (**Figures 8H–J**), further confirming the essential role of TFF1 in maintaining the malignant phenotype of lung cancer cells.

**Figure 9:**
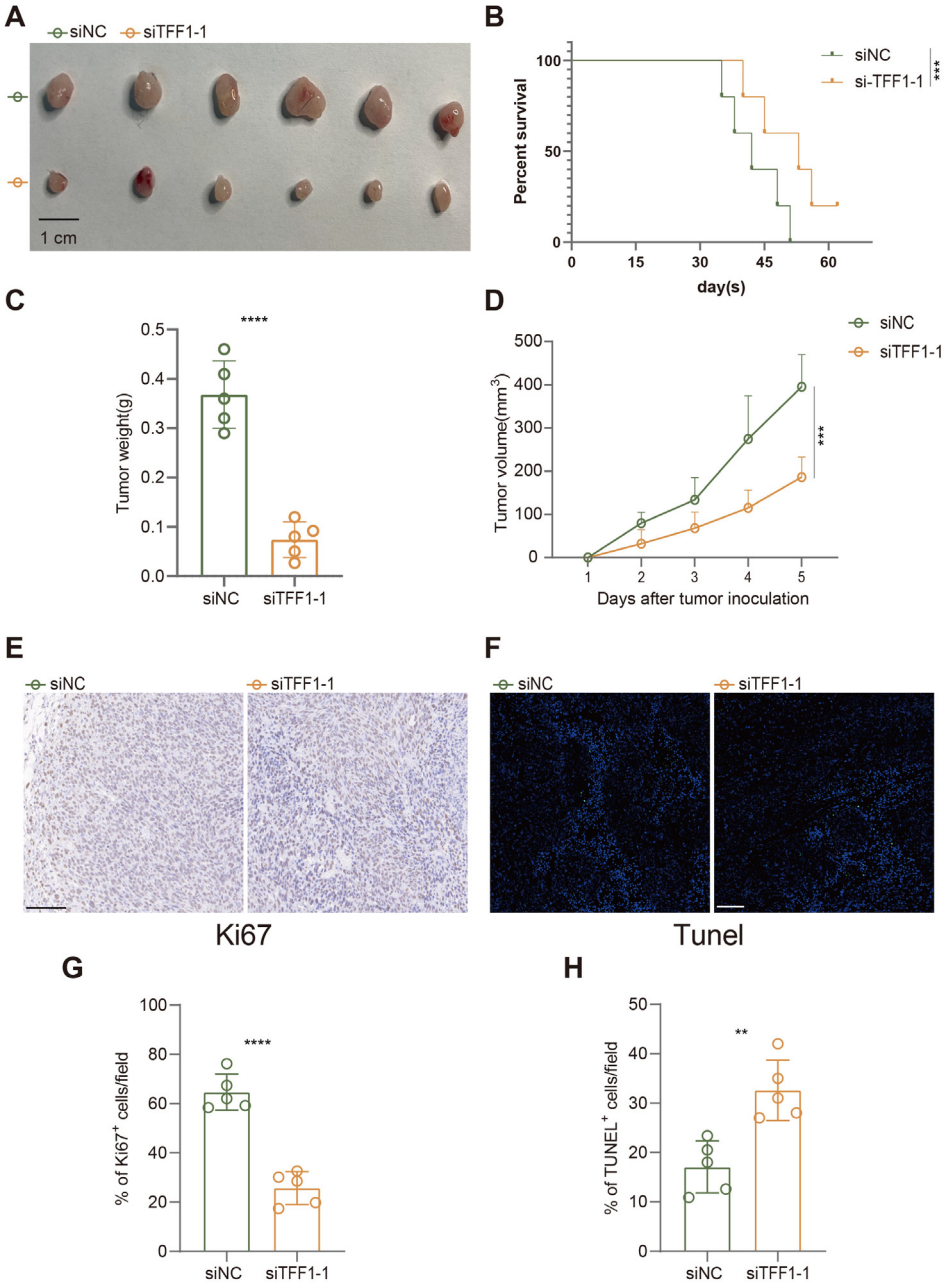
Effects of TFF1 knockdown on tumor growth, survival, and apoptosis in a subcutaneous tumor model in mice. **(A)** Subcutaneous tumors after injection of siTFF1-1 and siNC. **(B)** Kaplan-Meier survival curves show that the survival rate of mice in the siTFF1-1 group was significantly higher than that in the control group (siNC). **(C)** Tumor weight was significantly reduced after TFF1 knockdown. **(D)** TFF1 knockdown significantly inhibited the increase in tumor volume. **(E)** Ki67 immunohistochemical staining shows that cell proliferation was significantly reduced in the siTFF1-1 group compared to the control group. **(F)** TUNEL fluorescent staining shows a significantly higher proportion of apoptotic cells in the siTFF1-1 group compared to the control group. **(G, H)** Quantified percentages of Ki67-positive **(G)** and TUNEL-positive **(H)** cells per field. Data are presented as mean ± standard deviation. **p < 0.01, ***p < 0.001, ****p < 0.0001 indicate statistically significant differences compared to the siNC group.

In the established subcutaneous tumor model in C57BL/6 mice, we further evaluated the impact of TFF1 knockdown on tumor growth. As shown in the figures, subcutaneous tumors formed in mice injected with TFF1-knockdown LEWIS cells were significantly smaller than those in the control group (**Figures 9A, D**), and the tumor weight was also markedly reduced (**Figure 9C**). Additionally, the survival rate of mice in the TFF1 knockdown group was notably higher (**Figure 9B**), indicating that TFF1 knockdown significantly prolongs survival. Immunohistochemical analysis showed a substantial reduction in the proportion of Ki67-positive cells in tumors from the TFF1 knockdown group (**Figures 9E, G**), suggesting that the proliferative capacity of the tumor cells was suppressed. TUNEL staining results indicated a significant increase in the proportion of apoptotic cells in the TFF1 knockdown group (**Figures 9F, H**), further corroborating the critical role of TFF1 in tumor cell survival. These findings indicate that TFF1 knockdown not only suppresses the malignant phenotype of lung cancer cells *in vitro* but also significantly inhibits tumor growth and progression *in vivo*, highlighting its potential therapeutic value.

## Discussion

Malignant tumors are characterized by extensive global reprogramming of epigenetic patterns, including the gain or loss of DNA methylation and alterations in histone marks (49). Elucidating the network of epigenetic factors aids in understanding the mechanisms of interaction between genetic and epigenetic changes, thereby offering new therapeutic strategies for malignant tumors (50, 51).

Identifying tumor-driving cancer genes is crucial for understanding the pathways and gene functions in both normal and cancerous tissues (52–55). This identification is also a necessary prerequisite for developing cancer biomarkers and targeted therapies (49, 56). Epigenetic changes are considered one of the key hallmarks of tumors, driven by chromatin regulators (CRs) (21, 57). Chromatin remodeling refers to the dynamic changes in chromatin structure involved in genetic and epigenetic regulation, which impact gene expression (58). This remodeling can be achieved through modifications such as histone acetylation and methylation (4, 59, 60). Chromatin remodeling proteins alter the interactions between DNA and histone octamers on nucleosomes, facilitating the movement, rearrangement, and reorganization of chromatin fibers. Consequently, this changes the chromatin’s compaction and three-dimensional structure, thereby influencing gene expression (61–63).

Growing evidence underscores the critical role of epigenetic modifications in the initiation and progression of various cancers, including lung adenocarcinoma (LUAD) (64, 65). However, the significance of chromatin remodeling-related genes and their impact on lung cancer remains unclear. Our study employed a novel artificial intelligence framework comprising 429 machine learning algorithms and used a 10-fold cross-validation framework. We integrated multiple algorithms, including random survival forest (RSF), elastic network (Enet), Lasso, Ridge, stepwise Cox, CoxBoost, partial least squares regression for Cox (plsRcox), supervised principal components (SuperPC), generalized boosted regression modeling (GBM), and survival support vector machine (survival-SVM). By combining these algorithms with differentially expressed chromatin remodeling genes in cancerous and adjacent non-cancerous tissues of LUAD patients, we constructed a prognostic signature called CRRS. Using the optimal cut-off value for CRRS, we divided LUAD patients into high- and low-risk groups and employed Kaplan-Meier survival analysis to evaluate the predictive ability of CRRS on patient survival. The results demonstrated that the constructed CRRS effectively predicted the survival of LUAD patients. This finding was reliably validated across external datasets, including GSE42127, GSE31210, GSE30219, GSE29016, GSE26939, and GSE13213.

Subsequently, we used the C-index to evaluate the model’s performance. We validated the significance and expression levels of the 29 CR-related modeling genes in the CRRS across TCGA-LUAD and multiple GEO datasets (GSE42127, GSE31210, GSE30219, GSE29016, GSE26939, and GSE13213), with references verified using PMID numbers. The C-index results indicated that our model has good predictive performance. Principal component analysis further showed clear stratification between high- and low-risk LUAD patients (**Figure 4C**).

To explore the generalizability of CRRS’s predictive performance, we conducted clinical prognosis analyses using the TCGA database on various tumor samples, including UVM, THCA, SKCM, SARC, PRAD, PCPG, and PAAD. The results demonstrated that CRRS not only predicts the clinical prognosis of LUAD patients but also performs well in other cancers. Enrichment analysis of differential expression between high and low-risk groups revealed that CRRS might be involved in biological processes such as G2M checkpoint, E2F targets, MYC targets V2/V1, and DNA repair.

Additionally, using immune infiltration analysis algorithms such as TIMER, CIBERSORT, CIBERSORT-ABS, QUANTISEQ, MCPCOUNTER, XCELL, and EPIC, we found that there are differences in immune cell infiltration between high and low-risk CRRS groups. These differences include T cells, B cells, myeloid dendritic cells, macrophage M1, and macrophage M2. Further analysis of costimulatory and coinhibitory molecules (Co-stm and Co-inb), ligands and receptors involved in intercellular communication (e.g., TNFSF9, TNFRSF18), cell adhesion molecules, and antigen presentation molecules (e.g., ITGB2, HLA-DRB5) revealed differential expression between high and low-risk groups. These findings suggest potential differences in immune checkpoint inhibitors between CRRS high and low-risk patients, providing a foundation for further research into the mechanisms of immune checkpoint inhibitors and the identification of new therapeutic targets.

Furthermore, enrichment analysis results revealed that certain gene sets are significantly enriched in high-risk patients, such as MMD4_TARGETS_NEUROEPITHELIUM_DN, mitotic cell cycle process, and cell cycle. In contrast, other gene sets, like HP_ABNORMALITY_OF_THE_AXILLARY_HAIR, are enriched in low-risk patients. Specific functional categories, such as mitotic cell cycle process and cell cycle, were notably enriched in the network, underscoring their importance in prognosis. These findings suggest that these gene sets may play a crucial role in prognosis. This knowledge contributes to a better understanding of the molecular mechanisms of LUAD and provides a reference for clinical prognosis evaluation and treatment strategies.

Finally, our experimental results suggest that TFF1, one of the modeling genes, may serve as a potential therapeutic target for LUAD. Knockdown of TFF1 inhibited the proliferation of lung cancer cells, reduced colony formation efficiency, and increased apoptosis rates. *In vivo* studies further demonstrated that TFF1 knockdown slowed subcutaneous tumor growth in mice, decreased the proportion of Ki67-positive cells, and increased the number of TUNEL-positive cells. These findings suggest that TFF1 could be a promising target for lung cancer treatment and provide a foundation for further research.

However, there are some limitations to the current study that need to be addressed. First, the algorithms used in constructing the CRRS were based entirely on publicly available datasets, such as TCGA-LUAD, which may introduce biases related to data collection and curation. While these datasets are robust and widely used, the lack of direct validation using experimental or clinical data from our institution limits the broader applicability of our findings. Future studies should focus on validating the CRRS in larger, more diverse cohorts, including patient-derived datasets, to ensure its generalizability across populations with varying clinical and genetic backgrounds.

Overall, this study explores the global reprogramming of epigenetic patterns in malignant tumors, with a particular focus on the role of chromatin remodeling in lung adenocarcinoma (LUAD). By utilizing a novel artificial intelligence framework and multiple machine learning algorithms, the research constructed a prognostic signature, CRRS, comprising 429 algorithms, which was validated using multi-omics data. The results showed that CRRS could effectively predict the survival of LUAD patients and was validated across several independent datasets. Further analysis indicated that high-risk patients are significantly enriched in biological processes such as the cell cycle and DNA repair, and there are differences in immune cell infiltration and responses to immune checkpoint inhibitors. This study highlights the importance of chromatin remodeling-related genes in the prognosis of LUAD, providing a foundation for understanding its molecular mechanisms and developing new therapeutic strategies. However, there are limitations, as the current algorithms are based on public data and require further validation with self-tested data.

## Conclusion

This study explores the global reprogramming of epigenetic patterns in malignant tumors, with a particular focus on the role of chromatin remodeling in lung adenocarcinoma (LUAD). By utilizing a novel artificial intelligence framework and multiple machine learning algorithms, the research constructed a prognostic signature, CRRS, comprising 429 algorithms, which was validated using multi-omics data. While the CRRS model holds promise for improving patient stratification and guiding treatment decisions, it is important to acknowledge its limitations. The reliance on publicly available datasets for algorithm training introduces potential biases, and further validation with self-generated clinical data is needed to confirm its broader applicability. Future research should focus on expanding the validation of CRRS in diverse patient cohorts and exploring the therapeutic potential of TFF1 in clinical settings. In conclusion, this study highlights the importance of chromatin remodeling in LUAD prognosis and identifies TFF1 as a promising therapeutic target. These findings provide a foundation for the development of more personalized treatment strategies and open new directions for research into chromatin regulator-related therapies.

## Data Availability

Publicly available datasets were analyzed in this study. This data can be found here: https://www.jianguoyun.com/p/Dbsa4vEQjNnsDBj6y9MFIAA.
